# Efficacy and Safety of Anti-SARS-CoV-2 Antiviral Agents and Monoclonal Antibodies in Patients with SLE: A Case-Control Study

**DOI:** 10.3390/biom13091273

**Published:** 2023-08-22

**Authors:** Giuseppe A. Ramirez, Maria Gerosa, Chiara Bellocchi, Daniel Arroyo-Sánchez, Chiara Asperti, Lorenza M. Argolini, Gabriele Gallina, Martina Cornalba, Isabella Scotti, Ilaria Suardi, Luca Moroni, Lorenzo Beretta, Enrica P. Bozzolo, Roberto Caporali, Lorenzo Dagna

**Affiliations:** 1 Unit of Immunology, Rheumatology, Allergy and Rare Diseases, IRCCS Ospedale San Raffaele, Via Olgettina 60, 20132 Milan, Italy; darroyo@salud.madrid.org (D.A.-S.); asperti.chiara@hsr.it (C.A.); gallina.gabriele@hsr.it (G.G.); moroni.luca@hsr.it (L.M.); bozzolo.enrica@hsr.it (E.P.B.); dagna.lorenzo@hsr.it (L.D.); 2 Faculty of Medicine, Università Vita-Salute San Raffaele, Via Olgettina 58, 20132 Milan, Italy; 3 Department of Clinical Science of Community Health, Research Center for Adult and Pediatric Rheumatic Diseases, Università degli Studi di Milano, Via Festa del Perdono 7, 20122 Milan, Italy; maria.gerosa@unimi.it (M.G.); lorenza.argolini@hotmail.it (L.M.A.); martina.cornalba@unimi.it (M.C.); isascotti@hotmail.com (I.S.); ilaria.suardi@unimi.it (I.S.); roberto.caporali@unimi.it (R.C.); 4 Unit of Rheumatology, ASST Gaetano Pini-CTO, Piazza Cardinale Andrea Ferrari 1, 20122 Milan, Italy; 5 Referral Center for Systemic Autoimmune Diseases, Fondazione IRCCS Ca’ Granda Ospedale Maggiore Policlinico di Milano, Via Francesco Sforza 35, 20122 Milan, Italy; chiara.bellocchi@unimi.it (C.B.); lorberimm@hotmail.com (L.B.); 6 Department of Clinical Science of Community Health, Section of Internal Medicine, Università degli Studi di Milano, Via Festa del Perdono 7, 20122 Milan, Italy; 7 Department of Immunology, Hospital Universitario 12 de Octubre, Av de Córdoba, 28041 Madrid, Spain; 8 Department of Immunology, Instituto de Investigación Biomédica, Hospital Universitario 12 de Octubre, Av de Córdoba, 28041 Madrid, Spain

**Keywords:** systemic lupus erythematosus, COVID-19, SARS-CoV-2, antivirals, monoclonal antibodies

## Abstract

Severe acute respiratory syndrome coronavirus 2 (SARS-CoV-2)-related disease (COVID-19) has spread pandemically with high rates of morbidity and mortality. COVID-19 has also posed unprecedented challenges in terms of rapid development of pharmacological countermeasures to prevent or contrast SARS-CoV-2 pathogenicity. Anti-SARS-CoV-2 antiviral agents and monoclonal antibodies have been specifically designed to attenuate COVID-19 morbidity and prevent mortality in vulnerable subjects, such as patients with immune-mediated diseases, but evidence for the safe and effective use of these drugs in this latter population group is scarce. Therefore, we designed a retrospective, multicentre, observational, case-control study to analyse the impact of these treatments in COVID-19 patients with systemic lupus erythematosus (SLE), a paradigmatic, multi-organ autoimmune disease. We identified 21 subjects treated with antivirals and/or monoclonal antibodies who were matched with 42 untreated patients by age, sex, SLE extension and duration. Treated patients had higher baseline SLE disease activity index 2000 scores [SLEDAI-2K median (interquartile range) = 4 (1–5) vs. 0 (0–2); *p* = 0.009], higher prednisone doses [5 (0–10) mg vs. 0 (0–3) mg; *p* = 0.002], and more severe COVID-19 symptoms by a five-point World Health Organisation-endorsed analogue scale [1 (0–1) vs. 0 (0–1); *p* < 0.010] compared to untreated patients. There was no difference between groups in terms of COVID-19 outcomes and sequelae, nor in terms of post-COVID-19 SLE exacerbations. Three subjects reported mild adverse events (two with monoclonal antibodies, one with nirmatrelvir/ritonavir). These data suggest that anti-SARS-CoV-2 antivirals and monoclonal antibodies might be safely and effectively used in patients with SLE, especially with active disease and more severe COVID-19 symptoms at presentation.

## 1. Introduction

Severe acute respiratory syndrome coronavirus 2 (SARS-CoV-2) is a recently emerging viral pathogen associated with multi-organ disorder (COVID-19) with prominent involvement of the respiratory tract. Delayed and dysfunctional anti-viral responses due to inborn or acquired immune defects constitute the core of COVID-19 pathogenesis, and have accounted for the high rates of morbidity and mortality observed in the general population during the first phases of the COVID-19 pandemic [1,2,3]. At that time, exposure of the immune system to unprecedented antigenic stimuli, along with SARS-CoV-2-specific mechanisms of evasion of the innate immune response, favoured systemic viral spread and misdirected and/or excessive activation of inflammation, ending in potentially life-threatening organ damage [4,5]. Later in the course of the pandemic, long-term sequelae of the SARS-CoV-2 infection, including exacerbation or a new onset of immune disorders, emerged as a significant cause of morbidity in survivors of acute COVID-19 [6]. Therefore, effective modulation of the immune response to SARS-CoV-2 has constituted the main goal of most anti-COVID-19 preventive and therapeutic treatment strategies. Vaccination has been the main game changer in this setting, especially for vulnerable subjects such as patients with immune-mediated diseases [7]. Nonetheless, patients with pre-existing defects in the deployment of the immune response—due to treatments or intrinsic disease-specific mechanisms—had relatively lower rates of humoral and cellular responses to vaccination and a slightly higher risk of developing symptomatic COVID-19, along with its sequelae, especially in cases of higher disease activity [8]. Furthermore, evidence from some studies also suggested that immunocompromised hosts might have shown impaired and delayed SARS-CoV-2 clearance, possibly leading to inadvertent facilitation of viral spread and generation of novel variants [9,10,11,12]. Patients with systemic lupus erythematosus (SLE), a paradigm autoimmune disorder, constituted a group of particular concern due to coexisting alterations in the innate and adaptive immune response as distinctive traits of its pathogenesis. Recent evidence has specifically revealed that a relevant fraction of patients with SLE bear anti- type I interferon (IFN-I) antibodies with a potential protective role towards SLE progression, but conferring enhanced susceptibility to severe COVID-19 [13]. Patients with SLE taking corticosteroids were also shown to be at increased risk of symptomatic COVID-19 and with a reduced response to vaccination [14].

To address the potential residual risk of severe or long-resolving COVID-19 in vulnerable subjects, targeted anti-SARS-CoV-2 treatments, including antivirals and monoclonal antibodies, have been developed or repurposed (in parallel with prophylactic treatments with vaccination) and rapidly approved for clinical use [15]. Stabilised chimeric SARS-CoV-2 spike protein domains bearing conserved conformational epitopes usually hidden to the immune system have also been proposed as platforms for the development or new vaccines or decoy spike components to be employed to treat COVID-19, but unfortunately they have not yet been developed at a clinical level [16]. Among antivirals, remdesivir, a nucleotide analogue originally developed for hepatitis C, Ebola and Marburg virus, was one of the first agents tested in COVID-19 clinical trials, yielding contrasting results [17,18,19,20]. Eventually, accumulating knowledge on COVID-19 pathophysiology and temporal dynamics showed that the drug might have been selectively effective in the very early phases of the infection, while being of questionable utility in later stages [21,22,23]. Molnupiravir, another small-molecule interfering with viral replication, received emergency approval by drug regulatory authorities based on the encouraging results of early clinical trials in patients with mild to moderate COVID-19 at risk of deterioration [24]. The drug was eventually refused market authorisation due to lack of sufficient evidence of efficacy in reducing hospitalisations, despite a possible benefit in all-cause mortality [25]. Ritonavir-boosted nirmatrelvir (nirmatrelvir/ritonavir) was specifically developed for coronaviridae and showed efficacy in preventing progression from mild/moderate to severe COVID-19 in at-risk subjects, despite potential concerns about the risk of toxic interactions with other drugs [26,27]. Anti-viral neutralising antibodies encompass monoclonal antibodies, recombinant single-chain variable region fragments, antigen binding fragments (Fab) or the so-called nanobodies; that is, single-domain antibodies [28,29]. Only monoclonal antibodies have been pharmacologically developed to clinical use for COVID-19 so far. Anti-SARS-CoV-2 monoclonal antibodies were developed from the sera of patients surviving COVID-19 and/or the 2003 SARS virus, and from murine disease models in a quest for novel prophylactic and therapeutic strategies to apply in vulnerable subjects with COVID-19 [30]. These agents target the receptor binding domain of the SARS-CoV-2 spike protein (which is fundamental for virus entry into host cells) and are modified in their Fc portion to potentiate opsonisation and reduce the antibody-dependent enhancement of viral spread [31]. Rapid generation of mutations in the spike protein amidst the emergence of new SARS-CoV-2 variants prompted the need for constant updates in the development of monoclonal antibodies. A combination of antibodies, such as in the case of bamlanivimab/etesivimab, casirivimab/imdevimab, and tixagevimab/ cilgavimab, or a selection of pan-coronavirus antibodies such as for sotrovimab, constituted additional countermeasures to prolong the timeframe of potential utility of these drugs for COVID-19 [31,32]. In parallel with clinical trials, real-life studies confirmed the relatively favourable efficacy and safety profile of these agents in the general population, at least in older subjects [33,34,35]. Nonetheless, very limited evidence exists regarding patients with immune-mediated diseases [36,37,38]. Therefore, we set up a retrospective multi-centre case-control study focusing on patients with SLE and COVID-19, analysing the impact of targeted anti-SARS-CoV-2 agents both on COVID-19 and the SLE course.

## 2. Materials and Methods

According to the 2012 SLE International Collaborating Clinics (SLICC) criteria, patients with SLE who developed COVID-19 between February 2020 and December 2022 were identified by patient chart review in a three-centre cohort constituting the SMILE, Milan Lupus Consortium (IRCCS Ospedale San Raffaele, Centre 1, ASST Gaetano Pini CTO, Centre 2, and Policlinico di Milano, Centre 3). This multicentre cohort represents approximately 10% of the total SLE population for the region of Lombardy and 2% of the Italian SLE population, according to recent SLE prevalence and general population demographics estimates [39]. Among these patients, subjects who had been treated with either anti-SARS-CoV-2 antivirals and/or monoclonal antibodies were age-, sex-, SLE extension- (number of British Isles Lupus Assessment Group index domains with current or previous involvement) and SLE duration-matched 1:2, with control subjects not having received these treatments. Patients receiving high-dose corticosteroids or biological agents other than SARS-CoV-2-targeted monoclonal antibodies to treat COVID-19 were excluded (Supplementary Figure S1). Upon written informed consent, case and control subjects were enrolled in an observational study focusing on COVID-19 and the post-COVID-19 SLE course. The study was conducted in accordance with the Declaration of Helsinki and approved by local Institutional Review Boards: Panimmuno research protocol (approval number 22/INT/2018 by the San Raffaele Hospital Institutional Review Board); MLC protocol (approval number 0002450/2020 by the Comitato Etico Milano Area2 and 84/INT/2019 by the San Raffaele Hospital Institutional Review Board). Complete epidemiological data on the prevalence of COVID-19 within the study timeframe were available for Centre 1 and Centre 2 only.

Baseline data included demographics, SLE serological profile, clinical manifestations and treatments in SLE history and at the time of COVID-19. SLE disease activity at the time of COVID-19 development was assessed through the SLE Disease Activity Index 2000 (SLEDAI-2K) and damage through the SLICC/American College of Rheumatology Damage Index (SDI). COVID-19 baseline information encompassed the number of previous anti-COVID-19 vaccine doses and time from the last dose, along with COVID-19 symptoms at presentation. COVID-19 severity was measured through a 0–4 analogue scale endorsed by the World Health Organisation (WHO) [40]. Briefly, positive SARS-CoV-2 testing without symptoms of lower respiratory tract involvement corresponded to stage zero; patients with fever, cough or mild dyspnoea with signs of lower respiratory tract involvement but without signs of respiratory insufficiency were classified as stage 1; patients with initial signs of respiratory insufficiency, such as increased respiratory rate and/or reduced peripheral oxygen saturation were classified as stage 2; stage 3 corresponded to severe respiratory insufficiency requiring advanced oxygen support; stage 4 consisted of life-threatening disease with signs of multi-organ failure.

Follow-up data encompassed duration of COVID-19 symptoms and time to SARS-CoV-2 viral load negativisation, along with COVID-19-related complications. Complications encompassed death, hospitalisation and occurrence of any COVID-19-related manifestation requiring additional clinical assistance or treatment after the onset of COVID-19, including long COVID-19 [41]. We also recorded the time and characteristics of post-COVID-19 SLE flares from COVID-19 onset to the last rheumatological evaluation. We defined a disease flare as the occurrence of a new disease manifestation, or a worsening of pre-existing manifestations requiring immunosuppressive treatment escalation. The incidence rates of post-COVID-19 flares were compared with pre-pandemic historical data collected at Centre 1 between 2015 and 2019 using an in-house dedicated software [42]. Adverse events that were potentially related to antivirals and monoclonal antibodies were also recorded.

Statistical analyses were performed with Statacorp STATA^®^ version 15.0. Categorical data were compared among groups by using the chi-square test with Fisher’s exact correction. Continuous variables were reciprocally correlated by using the Spearman’s test and compared among groups through the Mann–Whitney U-test. Cox’s regression analysis was used to compare COVID-19 remission achievement and SLE flare trends among groups. Data are expressed as median (interquartile range, IQR) or percentages, unless otherwise specified.

## 3. Results

### 3.1. Demographics and SLE Status at Time of COVID-19 Diagnosis

We identified a total of 21 patients with SLE (19 women, two men) and COVID-19 who had been treated with antivirals (n = 15) or monoclonal anti-SARS-CoV-2 antibodies (n = 6) from February 2020 to December 2022. In the same timeframe, 39% of patients in Centre 1 and Centre 2 had COVID-19 at least once, with the first SARS-CoV-2 infection occurring during 2022 in 73% of cases, during 2021 in 11% of cases, and during 2020 in 16% of cases. Among the 21 treated subjects, seven patients received remdesivir, five nirmatrelvir/ritonavir, three molnupiravir, two bamlanivimab/etesivimab, two sotrovimab, one casirivimab/imdevimab and one an unspecified monoclonal antibody. Details on the treatment protocols are provided in Supplementary Table S1. One patient (a 49-year-old woman with a 21-year history of SLE and a high burden of comorbidity, yielding a baseline SDI of nine points) had also received prophylaxis with tixagevimab/cilgavimab before having COVID-19 and being treated with molnupiravir. SARS-CoV-2-targeted treatments were administered in dedicated COVID-19 outpatient clinics or at home under the supervision of patient general practitioners. Treatment initiation occurred after 2 (1–3) days from symptom onset. The median (IQR) age of treated patients at enrolment was 48 (34–56) years and the median (IQR) disease duration was 12 (7–21) years. These patients were matched with 42 patients with SLE and COVID-19 with similar demographics and disease characteristics (Table 1).

The majority of subjects had musculoskeletal, mucocutaneous and haematological involvement. More than 75% of patients had a positive ADNA profile and a history of low complement, while only a minority had positive anti-Smith antibodies. Antiphospholipid antibodies were part of the serological profile of 40% of subjects, with 10% of subjects being classified with antiphospholipid syndrome. In terms of treatment history, most subjects were exposed to hydroxychloroquine, one or more immunosuppressants and high-dose glucocorticoids. Most patients had received azathioprine at least once in their disease history. Nine subjects had received rituximab at least once, with the last rituximab infusion having occurred 12 (9–35) months before COVID-19 onset.

At the time of COVID-19 diagnosis, treated subjects had a median (IQR) SLEDAI-2K of 4 (1–5) points, which was significantly higher than the median (IQR) score of untreated patients [0 (0–2) points; *p* = 0.009]. Accordingly, treated subjects were taking higher prednisone-equivalent doses at time of COVID-19 diagnosis [5 (0–10) mg/day] than untreated subjects [0 (0–2.5) mg/day; *p* = 0.002] and showed a trend towards a higher frequency of concomitant immunosuppressant treatment (67% vs. 48%; *p* = 0.187]. Specifically, 8/21 treated patients were taking mycophenolate mofetil (vs. 7/42 untreated patients), 5/21 were taking azathioprine (vs. 9/42 untreated patients), 1/21 was taking MTX (vs. 3/42 untreated patients) and 2/21 were taking other immunosuppressants (vs. 1/42 untreated patients). There was no difference in the prevalence of subjects receiving immunomodulatory treatments with hydroxychloroquine (67% vs. 79%) and belimumab (33% vs. 31%) between the treated and untreated group. Chronic damage was numerically higher in treated [SDI = 1 (0–4) points] than in untreated patients [SDI = 0 (0–2); *p* = 0.060]. Demographics, disease duration, SLEDAI-2K and SDI at time of COVID-19 did not differ among recruiting Centres. Centre 2 had a higher prevalence of patients with a history of nephritis (9/13) compared to Centres 1 and 3 (8/50; *p* = 0.001). No other differences were observed in terms of SLE history.

### 3.2. COVID-19 Course during the Acute Phase

All enrolled subjects had mild to moderate COVID-19 at presentation, consistent with the clinical indications of SARS-CoV-2-targeted treatments. The most frequent features at presentation were upper respiratory tract symptoms such as rhinitis along with fever, while only 13% of patients had dyspnoea and less than 10% had confirmed COVID-19 pneumonia. Two thirds of patients had already received three or more vaccine doses (with mRNA-based preparations) at time of COVID-19 onset. The median (IQR) time from the last vaccine dose to the development of COVID-19 was 120 (45–210) days. The overall median (IQR) time from COVID-19 onset to symptom resolution was 5 (3–8) days, while the time to SARS-CoV-2 viral sequence negativisation was 10 (7–14) days.

COVID-19 severity at presentation was significantly higher in treated patients than in untreated patients [WHO class = 1 (0–1) vs. 0 (0–1); *p* < 0.010], despite no difference in terms of previous anti-SARS-CoV-2 vaccine doses and time from last dose (Table 2). Specifically, 14/21 (67%) treated subjects had WHO class ≥ 1, compared to only 14/42 (33%) of untreated subjects. Patient disease severity at presentation and symptom prevalence did not differ among recruiting Centres.

There was no significant difference between treated and untreated patients in terms of time to COVID-19 symptom resolution and viral clearance. COVID-19-related complications were numerically higher in untreated subjects, but no statistical significance was achieved. Complication prevalence did not differ significantly among Centres, although 6/7 events were recorded at Centre 1 over 34 patients. There was also no statistically significant difference in the number of COVID-19 related hospitalisations. All patients in the treated group survived, while one death was recorded in the control group. This patient was a 72-year-old woman with a history of concomitant myasthenia gravis, who became infected before the availability of vaccines and SARS-CoV-2-targeted treatments. Among treated subjects, no significant differences were identified between antivirals and monoclonal antibodies in terms of COVID-19 presentation and course.

Among patients treated with antivirals and/or monoclonal antibodies, three had at least one adverse event occurring during treatment. One patient developed skin rash and gastrointestinal symptoms three days after starting nirmatrelvir/ritonavir. This patient was a 56-year-old woman with a 29-year history of SLE with joint, constitutional and ophthalmological manifestations, who had developed COVID-19 with dyspnoea, fever, and upper respiratory symptoms while being on low dose prednisone (2.5 mg/day) for low disease activity (SLEDAI-2K = 2). Her total SDI score was four, indicating moderate damage accrual. She had no allergy history. Nirmatrelvir/ritonavir was precautionarily discontinued without further sequelae. She had no disease flares up to 290 days after COVID-19. Two patients had mild adverse events with monoclonal antibodies. One patient was a 31-year-old woman with a ten-year history of SLE with prominent musculoskeletal, mucocutaneous and haematological manifestations. She had moderate disease activity (SLEDAI-2K = 6) and was taking moderately high prednisone doses (12.5 mg/day) at time of COVID-19 diagnosis, which presented with stage 1 severity according to the WHO scale. The patient developed mild muscle cramps, which eventually spontaneously subsided. Five months after COVID-19 and apparent improvement in disease activity, she experienced an arthritic flare. Another patient was a 72-year-old woman with late-onset SLE (two-year disease history) presenting with fever and upper respiratory symptoms while off corticosteroids and with low disease activity (SLEDAI-2K = 2). She was treated in another hospital with unspecified monoclonal antibodies and developed chest pain, which eventually self-resolved. She had no eventual SLE flares until the end of the observation.

### 3.3. Post-COVID-19 Course

Long COVID-19 symptoms were developed by an equal proportion of subjects among the two groups (4/21 vs. 6/42, *p* = 0.719). SLE exacerbation occurred in 2/21 treated and 4/42 untreated patients with COVID-19 over a cumulative observation time of 4199 person-days and 11326 person-days, respectively, yielding flare incidence rates of 17.4 (95% confidence interval, 95%CI = 2.0–62.8) cases/100 person-years and 12.9 (95%CI = 3.5–33.0) cases/100 person-years, respectively. Pre-pandemic historical data were available for one of the three Centres involved in the study (Centre 1): from 2015 to 2019, 113 flares over 526.4 person-years had been recorded, yielding an incidence rate of 21.5 (95%CI = 17.7–25.1) flares/100 person-years. There was no statistically significant difference in flare rates among the two groups and pre-pandemic trends. No significant difference was identified between antivirals and monoclonal antibodies in terms of SLE outcomes.

## 4. Discussion

In this case-control study, we found that treatment with SARS-CoV-2-targeted antiviral agents and/or monoclonal antibodies was associated with favourable COVID-19 outcomes in patients with SLE, along with low rates of adverse events. Treated subjects showed 100% survival, relatively fast symptom resolution and viral clearance along with low rates of post-COVID-19 complications. These outcomes were comparable to those observed in patients with SLE and COVID-19 who did not receive targeted treatments, despite significantly more severe COVID-19 presentation in the treated group. Post-COVID-19 SLE flare rates were comparable between treated and untreated subjects, despite higher baseline disease activity scores in the treated group. Flare rates were also consistent with expected trends based on historical data [43]. Taken together, these results support a relatively safe and effective use of antivirals and anti-SARS-CoV-2 monoclonal antibodies in patients with SLE.

Due to the turbulent pace of the COVID-19 pandemic, limited comparable evidence exists in the literature. Indeed, the number of immunocompromised patients receiving anti-SARS-CoV-2 treatments was quite low in our and other cohorts, especially when considering that such patients were predicted to have a higher prevalence of risk factors fitting the criteria for antivirals and monoclonal antibodies [36]. However, consistent with this finding, rates of monoclonal antibody and antiviral use have also been lower than expected in the general population. Insufficient preparedness of health systems in terms of logistics, communication and healthcare personnel training, along with the rapid evolution of the pandemic have been claimed as potential explanations for failure to achieve a generalised introduction of these drugs in the common clinical practice, especially in the case of drugs requiring intravenous administration [36,44,45]. In accordance with these findings, no randomised controlled trial specifically assessing the role of these pharmacological agents in patients with disorders of the immune system has been published to date. The largest available studies focused on the potential role of prophylaxis with monoclonal antibodies in preventing COVID-19 [37,46,47]. More limited evidence has been published for the non-prophylactic use of monoclonal antibodies and for antivirals [32,38]. In addition, most studies on immunocompromised and rheumatic disease patients only provided outcome descriptions without assessing the COVID-19 course in comparator groups of untreated subjects. The most consistent evidence across published studies has been the overall good safety profile of antivirals and monoclonal antibodies, despite potential complex pharmacological interactions among antivirals and concomitant immunomodulating/immunosuppressant agents [36]. In line with these findings, we recorded only mild and infrequent adverse events among patients described in our series. More controversial data have been published with regard to antiviral and monoclonal antibody efficacy, possibly due to heterogeneity in pandemic timeframes and treatment protocols among different studies. Specifically, while early publications showed non-significant or even detrimental effects of SARS-CoV-2-targeted treatments in immunocompromised subjects [48,49,50,51,52,53,54,55], more recent works report more favourable outcomes [32,38,56]. Adding to this evidence, our data support the association between the use of antivirals and SARS-CoV-2 monoclonal antibodies and a favourable infection course in patients with mild to moderate COVID-19 in the context of SLE, while highlighting the importance of including inflammatory disease and immunosuppressive treatment burden in patient risk stratification.

At the time of analysis completion and manuscript writing, the global health emergency status secondary to the COVID-19 pandemic has been declared as ended by the WHO [57], prompting de-escalation of containment measures and epidemiological monitoring across countries. In this context, the significance of data acquired through this retrospective study might be not straightforwardly evident. However, multiple reasons support the potential strength and utility of our results for present and future clinical practice and research. First, as emphasised in the position statement by the WHO, downgrading the emergency status of the pandemic, “does not mean COVID-19 is over as a global health threat” due to the residual burden of morbidity associated to COVID-19, both in the general population and in vulnerable subjects [57]. Regarding this latter group, most recent reports still show that immunocompromised individuals bear a higher risk of short- and long-term complications secondary to COVID-19 [58,59], potentially suggesting the need to keep considering anti-SARS-CoV-2-targeted treatments as viable therapeutic options for patients deemed at higher risk of complications. Second, as patients with immune-mediated disorders were identified as vulnerable subjects since the early phases of the pandemic and therefore assigned to enhanced shielding measures [60,61], acquiring clinical information on the impact of SARS-CoV-2 first and recurrent infections in larger patient populations and for longer timespans might inevitably be delayed compared to the general population. Consistently, we observed that more than 70% of first episodes of COVID-19 occurred in the latest phases of the pandemic in our cohort, which implies that we still have relatively limited knowledge on the potential long-term sequelae of these events in the majority of subjects. While existing literature suggests that inflammatory viral triggers, either due to new infections or reactivation of latent viruses or endogenous retroviral elements have a role in SLE exacerbations [62,63,64], we know little about SARS-CoV-2 re-infections, and might still need to revert to more aggressive pharmacological strategies to control these events with the natural evolution of SARS-CoV-2 over time. Fourth, data on drug efficacy and safety in patients with immune-mediated disorders such as SLE might be of additional importance for future therapeutic applications. Indeed, remdesivir use in COVID-19 constitutes itself a clear example of drug repurposing, having originally been developed for hepatitis C, and later on applied to Ebola or the Marburg virus infection [35,65]. In a similar way, drugs developed for and/or applied to COVID-19 might be of future usefulness for novel coronaviridae or for other infectious agents. More attractively, it can be speculated that antiretroviral treatments might also find additional therapeutic niches in the control of endogenous retroviral elements as a way to prevent rheumatic disease flares [62,66].

Despite its potential relevance for the application of SARS-CoV-2-targeted treatments in the near and far future, the evidence provided in this study is still limited by a number of factors, including its relatively low sample size and statistical power (80% for WHO class intergroup comparison), preventing deeper considerations about individual drug efficacy and safety profiles. This limitation is also enhanced by the fact that patient treatments for COVID-19, along with those employed for the background inflammatory disorder, were quite heterogeneous. The low number of patients with SLE treated with antivirals and monoclonal antibodies likely mirrors similar trends in the general population of the same geographical area, especially in light of the relatively high representativeness of our multicentre cohort with regard to the local SLE population [39,44]. Although the vaccinal status was balanced between the treated and control group in terms of number of vaccine doses, we did not have information about inter-individual and inter-group variability in terms of the biological response to vaccination, which might have affected COVID-19 severity at presentation and during the disease course. Furthermore, we included patients developing COVID-19 at different stages of the pandemic, that is due to different SARS-CoV-2 variants, adding potential confounders to the assessment of SARS-CoV-2-targeted agent efficacy. At least part of the potential confounding effect of these limitations is, however, mitigated by the unprecedented inclusion of a control group of subjects with comparable demographics and general disease characteristics alongside the group of patients who received antivirals and anti-monoclonal antibodies. Further studies will still be needed to confirm the evidence emerging from our investigation.

## 5. Conclusions

In conclusion, this study shows that treatment with SARS-CoV-2-targeted antiviral agents and monoclonal antibodies associates with favourable COVID-19 outcomes and relatively low rates of adverse events in patients with SLE, possibly suggesting their non-redundant role in compensating for the detrimental effect of SLE-related risk factors such as high disease activity, chronic morbidity and corticosteroid treatment. Evidence for this study supports the potential use of these drugs to prevent clinical deterioration in patients with SLE and COVID-19, and might be of further utility for future drug repurposing in the setting of SLE and rheumatic disorders. Due to the relatively limited number of patients included in this study, additional research is warranted to consolidate these data and dissect potential differences in safety and efficacy profiles of individual antivirals and monoclonal antibodies as treatments for COVID-19 in the setting of SLE and immune-mediated diseases.

## Figures and Tables

**Table 1:** Demographics and general clinical features.

	Treated	Untreated
Number of subjects	21 (100)	42 (100)
Women: n (%)	19 (90)	38 (90)
Age (years): median (IQR)	48 (34–56)	44 (34–55)
Disease duration (years): median (IQR)	12 (7–21)	11 (6–21)
Disease characteristics (history): n (%)		
Musculoskeletal inv.	19 (90)	36 (86)
Mucocutaneous inv.	14 (67)	29 (69)
Nephritis	8 (38)	9 (21)
Neuropsychiatric SLE	5 (24)	6 (14)
Cardiopulmonary inv.	5 (24)	10 (24)
Haematological inv.	15 (71)	27 (64)
Constitutional symptoms	8 (38)	11 (26)
Gastrointestinal inv.	3 (14)	2 (5)
Ophthalmic inv.	3 (14)	5 (12)
Antiphospholipid syndrome	3 (14)	3 (7)
Serological features (history): n (%)		
Antiphospholipid antibodies	7 (33)	18 (43)
ADNA	16 (76)	32 (76)
aSm	1 (5)	7 (17)
Low complement	16 (76)	34 (81)
Treatment history		
HCQ ever: n (%)	18 (86)	40 (95)
MTX ever: n (%)	7 (33)	10 (24)
MMF ever: n (%)	11 (52)	14 (33)
AZA ever: n (%)	14 (67)	20 (48)
CyA ever: n (%)	4 (19)	2 (5)
CYC ever: n (%)	4 (19)	4 (10)
RTX ever: n (%)	5 (24)	4 (10)
Time from last RTX dose (months): median (IQR)	10 (6–24)	32 (11–57)
High dose glucocorticoids ever: n (%)	17 (81)	22 (52)

Abbreviations: ADNA: anti-DNA antibodies; aSm: anti-Smith antibodies; AZA: azathioprine; CyA: cyclosporine A; CYC: cyclophosphamide; HCQ: hydroxychloroquine; MMF: mycophenolate mofetil; MTX: methotrexate, RTX: rituximab.

**Table 2:** COVID-19 presentation and course.

	Treated	Untreated
Number of subjects	21 (100)	42 (100)
Number of vaccine doses: median (IQR)	3 (2–3)	3 (2–3)
Time from last vaccine administration (days): median (IQR)	150 (60–195)	120 (38–210)
COVID-19 features		
WHO class at presentation: median (IQR)	1 (0–1) **	0 (0–1)
Symptoms at presentation		
Dyspnoea: n (%)	5 (24)	3 (7)
Fever: n (%)	13 (62)	25 (60)
Upper Respiratory Symptoms: n (%)	19 (90)	34 (81)
GI symptoms: n (%)	1 (5)	2 (5)
Pneumonia: n (%)	3 (14)	3 (7)
COVID-19 course		
Time to symptom resolution (days): median (IQR)	5 (4–8)	7 (3–8)
Time to viral clearance (days): median (IQR)	10 (7–14)	10 (7–14)
Any complication: n (%)	1 (5)	6 (14)
Hospitalisations: n (%)	1 (5)	0 (0)
Deaths: n (%)	0 (0)	1 (3)

**: *p* < 0.010.

## Data Availability

Data supporting this publication are available upon reasonable request to the corresponding author.

## Supplementary Materials

The following supporting information can be downloaded at: https://www.mdpi.com/article/10.3390/biom13091273/s1, Supplemental Table S1: anti-SARS-CoV-2 targeted agent treatment protocols; Supplemental Figure S1: study flow-chart.
