# Supplementary material for: Efficacy and Safety of Anti-SARS-CoV-2 Antiviral Agents and Monoclonal Antibodies in Patients with SLE: A Case-Control Study
Source: Biomolecules. 2023 Aug 22;13(9):1273. doi: 10.3390/biom13091273 (PMC10527378; doi:10.3390/biom13091273)

Supplemental Material to

Efficacy and Safety of Anti-SARS-CoV-2 Antiviral Agents and Monoclonal Antibodies in Patients with SLE: A Case-Control Study

Giuseppe A. Ramirez, Maria Gerosa, Chiara Bellocchi, Daniel Arroyo-Sánchez, Chiara Asperti, Lorenza M. Argolini, Gabriele Gallina, Martina Cornalba, Isabella Scotti, Ilaria Suardi, Luca Moroni, Lorenzo Beretta, Enrica P. Bozzolo, Roberto Caporali and Lorenzo Dagna

Supplemental Table S1. Anti-SARS-CoV-2 targeted agent treatment protocols

	Route of administration	Dose and duration of treatment
Remdesivir	Intravenous	200 mg (load) on day 1, 100 mg on day 2 and 3
Nirmatrelvir (NIR)/ ritonavir (RIT)	Oral	150 mg NIR 2 tablets + 100 mg RIT 1 tablet bid for 5 days*
Molnupiravir	Oral	200 mg 4 tablets bid for 5 days
Bamlanivimab (BAM)/ etesivimab (ETE)	Intravenous	700 mg BAM + 400 mg ETE single dose
Sotrovimab	Intravenous	500 mg single dose
Casirivimab (CAS)/ imdevimab (IMD)	Intravenous	1200 mg CAS + 1200 mg IMD single dose

* One patient discontinued the drug after 3/5 days for an adverse event (see main text).

Supplemental Figure S1. Study flow-chart

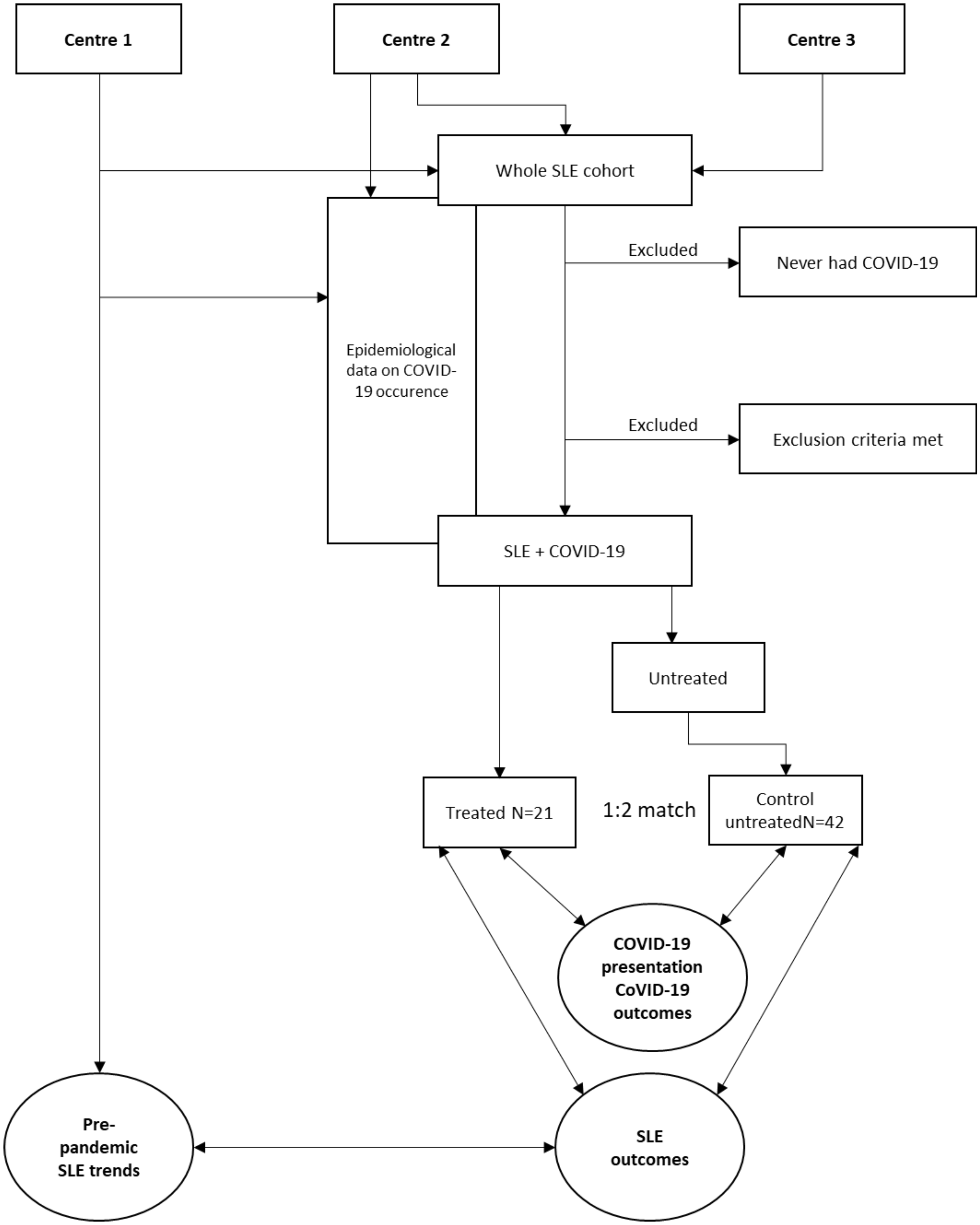

Supplement: Supplementary file 1 [file biomolecules-13-01273-s001.zip › biomolecules-2562254-supplementary.pdf]
